# Prognostic factors of penetrating neck trauma

**DOI:** 10.1590/S1808-86942011000100020

**Published:** 2015-10-19

**Authors:** José Cruvinel Neto, Rogério Aparecido Dedivitis

**Affiliations:** 1 Medical resident in generalsurgery, Municipal Hospital Dr. Fernando Mauro Pires da Rocha “Campo Limpo,” São Paulo, SP, Brazil; 2 Associate professor, UNILUS Lusíada Foundation. Physician

**Keywords:** wounds penetrating, neck injuries, penetrating

## Abstract

The neck is vulnerable to trauma because of vital structures and possible major injuries with high morbidity and mortality rates.

**Aim:** To evaluate the outcome in patients with penetrating cervical wounds.

**Methods:** The medical registries of 39 patients were analyzed retrospectively from 2001 to 2009. Penetrating wounds were defined as injuries that penetrated beyond the platysma muscle. Age, gender, etiology, wound site, injured structures, treatment, and outcome were analyzed. Fisher's exact test was adopted to establish the link between these variables and the outcome (discharge or death).

**Results:** Of 39 patients, 33 (84.62%) were men with a mean age of 28 years. The main cause was firearm projectiles - 19 (48.72%) cases; the most frequently affected zone was zone II - 29 (74.36%). Thirteen (33.3%) cases of hemodynamic instability were observed, and the average hospital stay was 14 (1-99) days. The main indication for surgical intervention was the presence of profuse hemorrhage, in eight (20.5%) cases. The main structures affected were the cervical veins (20.5%). There were eight (20.51%) deaths. Younger patients had a better prognosis.

**Conclusion:** The mortality rate was 20.51%. Patients below age 26 years had a better prognosis.

## INTRODUCTION

The neck is vulnerable to trauma because of the large number of vital structures in such a confined and small area. Thus, minor aggression may cause significant injury with high morbidity and mortality. The first documented case of treatment of neck trauma is attributed to the French surgeon Ambroise Paré (1510-1590), who ligated the jugular vein and carotid artery of a soldier that had suffered a bayonet injury.^1^

During the 1950s, a study of one hundred victims of penetrating neck would showed that the mortality was 6% when patients were operated immediately, and 35% were observed and operated later; early exploration was then established.^2,^^3^ In the 1970s, a selective approach was defended; patients were not operated immediately, but rather observed and monitored with exam.^4,^^5^ The reasons for the selective approach are: a high rate of negative cervicotomies, increased morbidity of early exploration, the risk of additional harm during routine exploration, the lower cost of selective exploration, the availability of endoscopy, angiography and other image methods (computed tomography), and the possibility of transferring patients to centers with more experienced professionals.^6^

The purpose of this study was to assess the causal factors, treatment, and outcome of patients with penetrating neck wounds, seen at a tertiary hospital.

## METHODS

A study was made of the registries of 39 patients with penetrating neck injury, seen at an emergency unit from July 2001 to July 2009, in which exploratory neck surgery was carried out. Penetrating wounds were defined as injuries with platysma violation.

The institutional review board of the institution approved this study (no. 038/2010).

The files of patients were made obtained by retrospectively reviewing the registries of the surgical theater. Patients with non-penetrating or blunt wounds were not included in this study.

Age, gender and etiology were surveyed. Wounds were separated according to the zones (I, II and III) suggested by Roon and Christensen.^3^ Zone I extends from the clavicles to the lower border of the cricoid cartilage; zone II is demarcated by the lower border of the cricoid cartilage up to the angle of the mandible; and zone III is the regions from the angle of the mandible to the base of the skull. The injured structures and the treatment were annotated; negative cervicotomies were those in which no injury of blood vessels, aerodigestive tract components, nerves, important muscles and glands were found. The outcome was either discharge from hospital or death during the hospital stay.

Frequency distribution was applied to describe the categorical variables, central tendency measures (mean and median), and variability (variation and standard deviation) for numerical variables. Fisher's exact test was applied to check associations among variables and the outcome. The significance level was 5% for all statistical tests. The Stata version 10.0 software was used for the statistical analyses.

## RESULTS

There were 33 male patients (84.62%) in the study sample (39 patients). Age ranged from 14 to 55 years; the mean age was 28 years. The main causes of penetrating neck injury were gun shot wounds, 19 cases (48.72%), and stab wounds, 20 cases (51.3%). The most frequently involved area was zone II (29 cases, 74.36%), followed by zone I (9 cases, 23.08%), and zone III (one case, 2.56%) (Table 1).

**Table 1:** Distribution of the series

Variable	Category / measures	Frequency (%) or measures
Age (years)	n	39
	Minimum - Maximum	14 - 55
	Median	26
	Mean	28
	Standard deviation	10,7
Gender	Male	33 (84,6)
	Female	6 (15,4)
Etiology	Gun shot wounds	19 (48,7)
	Stab wounds	20 (51,3)
BP + HR	Stable	26 (66,7)
	Unstable	13 (33,3)
Zone	I	9 (23,1)
	II	29 (74,4)
	III	1 (2,6)
Outcome	Death	8 (20,5)
	Discharge	31 (79,5)

There were 13 cases (33.3%) of hemodynamic instability. The mean hospital stay was 14 days (1 to 99 days). The main indication for surgical exploration was profuse hemorrhage in 8 cases (20.5%), followed by subcutaneous emphysema in 6 cases (15,4%) and expanding hematoma also in 6 cases (15.4%). The most commonly affected anatomical structures were the neck veins (20.5%), followed by neck arteries (10.3%), and the trachea (10.3%) (Table 2).

**Table 2:** Distribution of the series according to indication and investigation

Variable	Category	n (%)
Indication	Profuse hemorrhage	8 (20,5)
	Subcutaneous emphysema	6 (15,4)
	Expanding hematoma	6 (15,4)
	Neurologic injury	1 (2,6)
	Gun shot wound	6 (15,4)
	Stab wound	5 (12,8)
	Hemothorax	1 (2,6)
	Hemodynamic instability	3 (7,7)
	Pneumomediastinum	1 (2,6)
	Profuse hemorrhage + SC emphysema	1 (2,6)
	Neurologic injury + Instability	1 (2,6)
	Arteries	4 (10,3)
	Vertebrae	2 (5,1)
	Veins	8 (20,5)
	Trachea	4 (10,3)
	Pharynx and esophagus	6 (15,4)
	Thyroid	1 (2,6)
	Nothing	2 (5,1)
Investigation	Tongue Muscle injury	1 (2,6) 1 (2,6)
	Arteries + Veins	2 (5,1)
	Vertebrae + Pharynx and esophagus	1 (2,6)
	Veins +Trachea	1 (2,6)
	Veins+ Pharynx and esophagus	2 (5,1)
	Trachea + Thyroid	2 (5,1)
	Trachea + Larynx	1 (2,6)
	Veins + Trachea + Thyroid	1 (2,6)

Surgery consisted of vascular ligatures in 17 cases (34%); the internal jugular vein (6 cases, 12%) and the external jugular vein (6 cases, 12%) were the most frequently involved. Tracheal suture was done in 6 cases (12%), followed by thyroid suture (5 cases, 10%) and pharyngeal suture (5 cases, 10%). There were 8 deaths (20.51%); 31 patients (79.5%) were discharged from hospital. The mean age of survivors was lower (26 years) than that of the group of patients that died (Table 3).

**Table 3:** Distribution of the series according to the outcome

Variable	Category	Outcome Death Discharge		*p*-value
		n (%)	n (%)	
Age range (years)	≤ 18	1 (10,0)	9 (90,0)	0,653
	> 18	7 (24,1)	22 (75,9)	
	N	8	31	
Age (years)	Mínima - Máxima	18 - 55	14 - 50	0,0308 *
	Mediana	35	24	
	Média	35,8	26	
	Desvio padrão	12,0	9,5	
Gender	Masculino	8 (24,2)	25 (75,8)	0,313
	Feminino	0 (0,0)	6 (100,0)	
Etiology	FAF	4 (21,0)	15 (79,0)	> 0,999
	FAB	4 (20,0)	16 (80,0)	
BP + HR	Estável	5 (19,2)	21 (80,8)	> 0,999
	Instável	3 (23,1)	10 (76,9)	
Zone	I	3 (33,3)	6 (66,7)	NA
	II	4 (13,8)	25 (86,2)	
	III	1 (100,0)	(0,0)	

*p* value based on Fisher's exact test; * p value based on the Mann-Whitney U test.

## DISCUSSON

High comorbidity rates occur in penetrating neck injury because of the number of vital structures in a confined region. Neck wounds have deserved attention from public authorities because of increasing urban violence in major cities. Care should always be given to the airways in patients with penetrating or blunt neck trauma, as a safe airway - whether by orotracheal intubation, cricothyroidostomy or tracheostomy - is the main duty of the medical team towards these cases in an emergency ward.^7^ Penetrating neck injury is evidence of increased urban violence in recent times; trauma that used to be studied during war may now be analyzed in civilian settings.

Current recommendations are surgical exploration in unstable patients: hemodynamic instability, profuse hemorrhage, expanding hematomas, hissing/frothing or air, subcutaneous emphysema, hoarseness, and dysphonia.^8^, ^9^, ^10^ If platysma violation is present but patients are stable, with adequate airway permeability, respiratory and ventilator support, and stable circulation, may be assessed by several diagnostic methods: Doppler ultrasound, computed tomography, arteriography, and others; these procedures may help avoid unnecessary surgery.^11^, ^12^, ^13^

Males predominated in this study (33 cases, 84.62%); the mean age was 28 years (14 to 55 years). Other series have also reported a predominance of males, ranging from 71 to 91%, and mean ages ranging from 25 to 34.5 years.^1,^^7,^^13,^^14^

The most frequently affected region in our study was zone II (29 cases, 74.36%), followed by zone I (9 cases, 23.08%), and zone III (one case, 2.56%). In the literature, zone II is also the most frequently affected site (59 to 82%), followed by zone I (5 to 25%), and zone III (2 to 25%).^1,^^7,^^14,^^15^ The increased mortality is due mainly to patients with zone I wounds and vascular injuries.^7,^^14^ Patients with associated injuries, especially neurological and spinal lesions, also had higher morbidity and mortality rates.^11^

Etiologically, there were 19 cases of gun shot wounds (48.72%), followed by 17 cases of knife wounds (43.59%). In this regard, the literature presents a variety of percentages, with the rate of gun shot wounds ranging from 4 to 75.5%, and the rate of knife wounds ranging from 20.7 to 95.7%.

Patients with potentially life-threatening injuries are being better managed because of advances in care and stabilization, better and more available diagnostic methods, interventional radiology, and improved surgical techniques. Surgery is not mandatory for all cases of penetrating neck injuries; it is not satisfactory because of a high rate of negative explorations. Thus, it is important to select patients carefully, provide adequate stabilization, and study each patient with image methods or observation to avoid unnecessary cervicotomies. Care should be individualized, and mandatory surgery may be done according to the resources of each institution.

The mortality rate was 20.51% (eight cases); this is high compared to other papers, in which mortality rates ranged from 1.5 to 7.5%.^1,^^7,^^13^, ^14^, ^15^, ^16^ The age group was younger (mean - 26 years) in patients that survived (*p* = 0,0308), which could suggest that younger patients have a higher probability of surviving. However, data on Table 3 showed no statistical difference in age association with death (cutoff point - 18 years) based on Fisher's exact test. Survivors were younger than patients that died; but other confounding factors may have influenced this observation.

## CONCLUSION

The mortality rate was 20.51%. The mean age of survivors was less than 26 years, lower than the mean age of the group of patients that died.
